# MERS-CoV: Understanding the Latest Human Coronavirus Threat

**DOI:** 10.3390/v10020093

**Published:** 2018-02-24

**Authors:** Aasiyah Chafekar, Burtram C. Fielding

**Affiliations:** Molecular Biology and Virology Research Laboratory, Department of Medical Biosciences, Faculty of Natural Sciences, University of the Western Cape, Private Bag X17, Robert Sobukwe Drive, Bellville 7535, South Africa; achafekar@uwc.ac.za

**Keywords:** human coronavirus, MERS-CoV, clinical features, upper respiratory tract infections, lower respiratory tract infections, respiratory viruses

## Abstract

Human coronaviruses cause both upper and lower respiratory tract infections in humans. In 2012, a sixth human coronavirus (hCoV) was isolated from a patient presenting with severe respiratory illness. The 60-year-old man died as a result of renal and respiratory failure after admission to a hospital in Jeddah, Saudi Arabia. The aetiological agent was eventually identified as a coronavirus and designated Middle East respiratory syndrome coronavirus (MERS-CoV). MERS-CoV has now been reported in more than 27 countries across the Middle East, Europe, North Africa and Asia. As of July 2017, 2040 MERS-CoV laboratory confirmed cases, resulting in 712 deaths, were reported globally, with a majority of these cases from the Arabian Peninsula. This review summarises the current understanding of MERS-CoV, with special reference to the (i) genome structure; (ii) clinical features; (iii) diagnosis of infection; and (iv) treatment and vaccine development.

## 1. Introduction

Given the diversity of animal coronaviruses, it was not surprising when another human coronavirus was isolated from a patient presenting with severe respiratory illness in June 2012. The 60-year-old man died as a result of renal and respiratory failure 11 days after admission to a hospital in Jeddah, Saudi Arabia [1]. The novel etiological agent was subsequently named Middle East Respiratory syndrome coronavirus (MERS-CoV) [2]. MERS-CoV is one of six known human coronaviruses that cause respiratory disease in humans and, with a mortality rate >35% [3], it is the first highly pathogenic human coronavirus to emerge since the global scare caused by the severe acute respiratory syndrome coronavirus (SARS-CoV) in 2003.

With the Kingdom of Saudi Arabia, the focal point of an ongoing MERS-CoV outbreak, the large number of religious pilgrims congregating annually in Saudi Arabia drastically increases the potential for the uncontrolled global spread of MERS-CoV infections [4]. In fact, infections have already been reported in more than 27 countries across the Middle East, Europe, North Africa and Asia [5,6,7,8].

This review focusses on the current information of MERS-CoV, with special reference to the genome structure, clinical features, diagnosis of infection and treatment and vaccine development. We also look at future prospects for MERS-CoV spread and prevention.

## 2. Genome Structure and Gene Functions

MERS-CoV, a lineage C *Betacoronavirus* (βCoVs), has a positive-sense single-stranded RNA (ssRNA) genome about 30-kb in size [9,10]. As of 2016, phylogenetic analysis of MERS-CoV has been done on 182 full-length genomes or multiple concatenated genome fragments, including 94 from humans and 88 from dromedary camels [11,12]. The MERS-CoV genomes share more than 99% sequence identity, indicating a low mutation rate and low variance among the genomes. MERS-CoV genomes are roughly divided into two clades: clade A, which contains only a few strains, and clade B, to which most strains belong [12].

**Figure 1:**
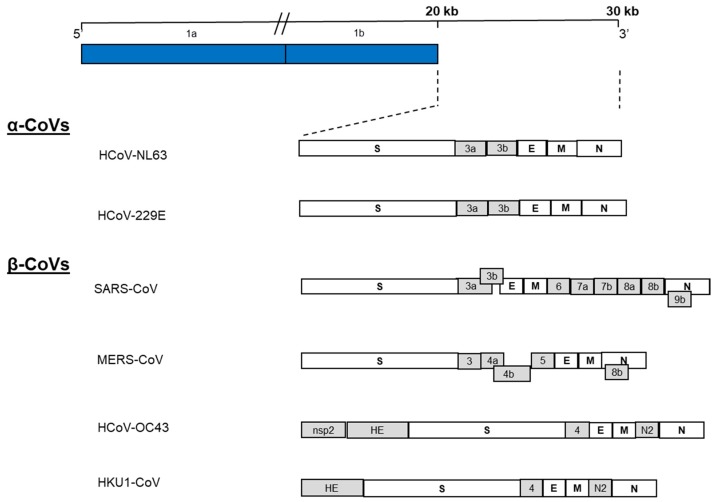
Schematic organization of human coronavirus (α and β CoVs) genomes. HCoVs genomes are 26 kb to 32 kb in size. At the 5′-end, overlapping reading frames 1a and 1b (blue) make up two-thirds of the genome. The remaining one third of the genome (expanded region) encodes for the structural (white) and accessory proteins (grey).

As with other CoV genomes, the first 5′ two-thirds of the MERS-CoV genome consist of the replicase complex (ORF1a and ORF1b). The remaining 3′ one-third encodes the structural proteins spike (S), envelope (E), membrane (M), and nucleocapsid (N), as well as five accessory proteins (ORF3, ORF4a, ORF4b, ORF5 and ORF8b) that are not required for genome replication (Figure 1), but are likely involved in pathogenesis [9,13,14,15,16,17] . The flanking regions of the genome contain the 5′ and 3′ untranslated regions (UTR) [13,14]. Typical of the coronaviruses, the MERS-CoV accessory proteins do not share homology with any known host or virus protein, other than those of its closely related lineage C βCoVs [12].

MERS-CoV structural and accessory protein-coding plasmids transiently transfected into cells showed that, while ORF4b was localised mostly in the nucleus, all of the other proteins (S, E, M, N, ORF3, ORF4a and ORF5) localised to the cytoplasm [18]. Furthermore, studies with MERS-CoV deletion-mutants of ORFs 3 to 5 are attenuated for replication in human airway-derived (Calu-3) cells [19], and deletion-mutants of ORFs 4a and 4b are attenuated for replication in hepatic carcinoma-derived (Huh-7) cells [16,20]. This clearly points to important putative roles for the MERS-CoV accessory proteins in viral replication, at least in an in vitro setting [21].

The principal response of mammalian cells to viral infection is the activation of the type I interferon (IFN)-mediated innate immune response through the production of type I IFNs (IFN-α and IFN-β). On the other hand, evasion of host innate immunity through IFN antagonism is a critical component of viral pathogenesis and is mediated by virus-encoded IFN antagonist proteins. Each protein blocks one or more key signaling proteins in the IFN and NF-κB pathways to enhance viral replication and pathogenesis [22,23,24,25]. Coronaviruses have similarly evolved these mechanisms to impede or bypass the innate immunity of their hosts at various levels, which ultimately contribute to coronavirus virulence. Various coronavirus proteins have previously been implicated in the disruption of signal transduction events required for the IFN response [26], often by interfering with the host’s type I interferon induction.

Evidence of MERS-CoV inducing type I IFN only weakly and late in infection (9–15) suggests that MERS-CoV has also evolved mechanisms to evade the host immune system. In fact, MERS-CoV M, ORF4a, ORF4b and ORF5 proteins are reported to be strong IFN antagonists [18]. Further studies, using the transient overexpression of MERS-CoV accessory protein ORF4a, ORF4b, and ORF5, show that the MERS-CoV accessory proteins inhibit both type I IFN induction [18,27,28] and NF-kappaB signaling pathways [28]. MERS-CoV ORF4a, a double-stranded RNA (dsRNA) binding protein [27], potentially acts as an antagonist of the antiviral activity of IFN via the inhibition of both the interferon production (IFN-β promoter activity, IRF-3/7 and NF-κB activation) and the ISRE promoter element signaling pathways [18]. MERS-CoV ORF4b, on the other hand, is an enzyme in the 2H-phosphoesterase (2H-PE) family with phosphodiesterase (PDE) activity. Even though MERS-CoV ORF4b is detected primarily in the nucleus of both infected and transfected cells [18,27,28], the expression levels of cytoplasmic MERS-CoV ORF4b are still sufficient to inhibit activation of RNase L, an interferon-induced potent antiviral activity [18,28]. MERS-CoV ORF4b is the first identified RNase L antagonist expressed by a human or bat coronavirus and provides a possible MERS-CoV mechanism for evasion of innate immunity by inhibiting the type I IFN and NF-kappaβ signaling pathways [16,28]. The MERS-CoV replicase proteins, including nsp1, nsp3 and nsp14, were also shown to interfere with the innate immune response signaling pathways through different mechanisms [21,29,30]. Evidently, MERS-CoV has developed various mechanisms to evade the host immune system.

## 3. Clinical Features

The median age of persons with laboratory-confirmed MERS-CoV infection is 49 years (range, <1–94 years); 65% of patients are males. The median time from illness onset to hospitalization is approximately four days, resulting in a median length of stay of 41 days [31]. Currently, among all patients, the morbidity rate is approximately 36% [3], with the median time from the onset of symptoms to death 11.5 days [32]. Chest radiography and computed tomography findings are generally consistent with viral pneumonitis and acute respiratory distress syndrome [33]. Laboratory findings include lymphopenia, thrombocytopenia and elevated lactate dehydrogenase levels [1,31,34,35,36,37,38,39], with some cases with a consumptive coagulopathy and elevations in creatinine, lactate dehydrogenase and liver enzymes [31,33,40].

The clinical spectrum of MERS-CoV infection ranges from asymptomatic infection [41,42,43] to rapidly progressive, acute respiratory distress syndrome, septic shock and multi-organ failure and death (see [32,44] for review of clinical spectrum). Initial symptoms are often nonspecific and patients report general malaise, including low grade fever, chills, headache, nonproductive cough, dyspnea, and myalgia [38,45]. Other symptoms can include sore throat and similar to SARS-CoV, MERS-CoV patients can also present with gastrointestinal symptoms such as anorexia, nausea and vomiting, abdominal pain and diarrhea [46,47,48]. Atypical presentations, including mild respiratory illness without fever and diarrheal illness, preceding the development of pneumonia have been documented [49]. Up to 50% of adult symptomatic patients require intensive care unit (ICU) treatment. These patients often show no sign of improvement and 40–70% typically require mechanical ventilation within the first week [32,41,50]. Renal replacement therapy is required for between 22–70% of critically ill patients [31,34,35,40,51], with the higher-end of the estimation possibly due to over-estimation as a result of hospital-acquired infections in patients with pre-existing renal disease [32,35].

MERS-CoV is linked with more severe disease in older people, people with weakened immune systems, and those with chronic diseases such as cancer, chronic lung disease and diabetes. The majority of patients who are hospitalized with MERS-CoV infection had chronic co-morbidities such as obesity, diabetes, hypertension, cardiovascular diseases or end-stage renal disease [40,52,53,54]. In fact, about 75% of patients testing positive for MERS-CoV have at least one co-morbid disease; fatal cases are more likely to have an underlying condition (86% among fatal cases vs. 42% among recovered or asymptomatic cases) [33].

Interestingly, MERS-CoV cases have been reported mainly in adults [55], with children rarely affected [56,57]. Even so, a recent case study of a MERS-CoV infected a 9-month-old child, newly diagnosed to have infantile nephrotic syndrome, showed complications that resulted in severe respiratory symptoms, multi-organ dysfunction and death [58]. In another study of 11 pediatric cases that tested positive for MERS-CoV, the two symptomatic patients had Down’s syndrome and cystic fibrosis, respectively, indicating that severe disease could potentially occur in children with serious underlying conditions [43]. Even with these reported pediatric cases, data on infection in children remain scarce, making it difficult to ascertain whether MERS-CoV is really a predominantly adult disease, or whether it often presents differently in children.

Simultaneous infection of the respiratory tract with at least two viruses is common in hospitalized patients, and although it is not clear whether these infections are more, or less, severe than single virus infections [59], mixed clinical features are commonly observed [60]; this makes clinical diagnosis unreliable and severely limit epidemiological studies of etiological agents. Similar to other respiratory viruses, MERS-CoV has been found in combination with a second respiratory virus, such as influenza A virus [48,61] respiratory syncytial virus, human parainfluenza virus 3 or human metapneumovirus [62,63,64]. MERS-CoV infected patients requiring mechanical ventilation also exhibited a similar co-infection profile with nosocomial bacterial infections including, *Klebsiella pneumoniae*, *Staphylococcus aureus*, *Acinetobacter species* and *Candida* species [47,65]. Preceding or concurrent viral respiratory tract infections can predispose the host to secondary co-infections from other microorganisms throughout the airway. The mechanisms by which viruses promote these superinfections are diverse and replete [66]. As yet, not much is known as to how MERS-CoV damages the airway and dysregulate the lung barrier function, which, in turn, supports the adherence and invasion of other pathogens into normally sterile sites within the respiratory tract.

Neuromuscular complications are not rare during MERS treatment, and could simply have been underdiagnosed previously [67]. The first cases of severe neurological syndrome, characterized by varying degrees of disturbed consciousness, ataxia, focal motor deficit and bilateral hyper-intense lesions were reported from a retrospective study of patients in ICU [68]. Another subsequent small retrospective study in Saudi Arabia reported that 25.7% of MERS patients developed confusion and 8.6% experienced some kind of seizure [69]. To date, other cases with central nervous system involvement, including one case of intracerebral haemorrhage as a result of thrombocytopenia, disseminated intravascular coagulation and platelet dysfunction, one case of critical illness polyneuropathy [70] and four cases that included Bickerstaff’s encephalitis overlapping with Guillain–Barre syndrome, intensive-care-unit-acquired weakness, or other toxic or infectious neuropathies [67], have been reported. Neurological complications in the latter study did not appear concomitantly with respiratory symptoms, but were delayed by 2–3 weeks [67].

MERS-CoV can be detected in respiratory tract secretions, with tracheal secretions and broncho-alveolar lavage specimens containing a higher viral load than nasopharyngeal swabs. The virus has also been detected in feces, serum and urine [48,71,72,73]. Virus excretion peaks approximately 10 days after the onset of symptoms [48], but viable viruses can be shed through respiratory secretions for up to 25 days from clinically fully recovered patients. In the healthcare setting, MERS-CoV has been isolated from environmental objects such as bed sheets, bedrails, IV fluid hangers and X-ray devices [7]. Another study also reported that MERS-CoV could survive for longer than two days at 20 °C and 40% relative humidity, confirming the risk of contact or fomite transmission in healthcare settings [74]. Viral RNA, on the other hand, is detected for up to five days on environmental surfaces following the last positive PCR from patients’ respiratory samples; RNA was detected in samples from anterooms, medical devices and air-ventilating equipment [7], but this is not necessarily indicative of viable virus.

## 4. Diagnosis of Infection

With no specific, reliable antiviral drug or vaccine approved for clinical use in MERS-CoV infections, rapid diagnostic tests are required to manage outbreaks of this virus. The first probe and primer sets for MERS-CoV detection by real-time RT-PCR were developed shortly after the initial reports of the disease [75,76]. Other early diagnostic tools included virus culture in Vero and LLCMK2 cells [1,77], but isolation and identification of viruses in cell culture is a slow, specialized and insensitive method [78].

Laboratory detection and confirmation of MERS-CoV infections has broadly included (i) molecular detection of MERS-CoV RNA; (ii) MERS-CoV antigen detection; or (iii) assays to identify a humoral response to prior MERS-CoV infection among humans [79] (Table 1). These assays have been used with varying degrees of success in terms of specificity, sensitivity, etc.

Currently, according to the WHO case definition, a positive real-time RT-PCR assay, targeting at least two different genomic regions, is used to confirm MERS-CoV infection (http://www.who.int/csr/disease/coronavirus_infections/case_definition/en/index.html) [80]. Of the different assay probes and primers sets used, those targeting ORF1a and upstream of the E gene show the highest sensitivity and remain the most widely used targets for MERS-CoV detection [75,81]. Additionally, a single positive assay result, confirmed by gene sequencing, can also be considered positive for MERS-CoV infection. A stumbling block here, though, is the fact that, when compared to real-time PCR, conventional RT-PCR typically generates lower quality sequence-ready templates [80,82,83,84,85,86], thereby limiting the usefulness of conventional RT-PCR in these single positive-sequencing assays.

Molecular tests can detect nucleic acids derived from MERS-CoV in clinical respiratory, serum, and stool specimens [81,87]. However, a major obstacle of conventional nucleic acid-based tests is that they require specialized molecular techniques and equipment, and are therefore not appropriate for point-of-care testing or bedside diagnosis. For this reason, for effective diagnosis and treatment of MERS-CoV infection, it is necessary to develop alternative methods that can be adapted to rapid and reliable clinical detection of MERS-CoV antigens. Here, the most appropriate tests would be assays detecting viral antigens or antibodies in the infected host [87].

## 5. Animal Models

Not only are laboratory animal species often used as models for human disease progression, they are also needed to study and evaluate novel therapies against emerging viruses [105]. Studies have shown that rabbits [106], ferrets [107], Syrian hamsters [108] and wild-type mice [109] are not suitable as models of MERS-CoV infection. More recently, four transgenic mouse models for MERS-CoV infection have been developed. In the first, a modified adenovirus expressing human DPP4 (huDPP4) is introduced intranasally to mice, which results in the expression of huDPP4 in all cells of the lung, not just those that natively express DPP4. In this model, mice show transient human DPP4 expression and mild lung disease. A concern with this model is that cells constitutively expressing DPP4 will be infected and the role of a broader infection of all cell types may change pathogenesis [110]. In the second model, a transgenic mouse was produced that expresses huDPP4 systemically. In this model, MERS-CoV infection leads to high levels of viral RNA and inflammation in the lungs, but, unfortunately, significant inflammation and viral RNA are also detected in the brains of infected mice, which represent a non-physiological expression pattern [111]. In the third model, a novel transgenic humanized mouse model was generated by replacing the mouse DPP4 coding sequence with that encoding huDPP4, ensuring correct physiological expression of huDPP4. Mice in this model show lung pathology consistent with the radiographic findings of interstitial pneumonia and significant lung disease as seen in humans infected with MERS-CoV. This suggests that this mouse model recapitulates pathological sequelae that are seen in MERS-CoV infection of humans. Importantly, unlike what is seen in other mouse models of MERS-CoV infection, virus replication and pathology in the huDPP4 mice are localized in the lungs and no inflammation develops in the brain, ensuring a more physiological accurate model of the human disease [112]. Finally, in 2016, Cockrell et al. generated a mouse model permissive for MERS-CoV infection, but with functional DPP4 immune function. Infecting this DDP4-chimeric mouse with a mouse-adapted strain of MERS-CoV, mimics MERS-CoV-induced respiratory disease without bystander neurologic disease [113]. 

Non-human primate models, including the rhesus macaque [114,115,116] and common marmoset [117] have also been reported as suitable animal models of MERS-CoV infection. Even though both species are susceptible to MERS-CoV infection, the extent of virus replication and severity of disease vary [105]. Rhesus macaques infected with MERS-CoV via intra-tracheal inoculation show clinical signs of disease, virus replication, histological lesions and neutralizing antibody production, indicating that this monkey model is suitable for studies of MERS-CoV infection [116]. On the other hand, the common marmoset reproduces several, but not all, features of MERS-CoV infection, and can potentially be used to evaluate novel therapies for human use [105,117].

## 6. Treatment and Vaccine Development

When no vaccines or specific antiviral drugs are available during an outbreak, nonspecific therapeutic interventions are often introduced to prevent severe morbidity and mortality. However, for this to be done effectively, a basic understanding of the pathogenesis of the disease is required and interventions are implemented based on observations of the clinical course of disease and complications. Due to the nature of many diseases, however, it is often not possible to assess, or systematically compare, different therapeutic approaches during an outbreak [118]. Similarly, in the case of MERS-CoV, it is necessary to monitor epidemic patterns and investigate the spread of infections to efficiently identify, control and prevent possible epidemics. For MERS-CoV infections, supportive care, which includes rest, fluids and analgesics are used, and mainly depends on the provision of organ support and management of complications [49,119,120]. Broad-spectrum antimicrobials, antivirals [121,122], interferon-α2b (96) and antifungals can be used to minimize the risk of co-infection with opportunistic pathogens [49,119].

Interestingly, combination treatment with ribavirin and interferons inhibits MERS-CoV replication in vitro, and it was also shown to improve clinical outcomes in MERS-CoV-infected non-human primates. However, this treatment in the rhesus macaques was initiated very soon after viral challenge (~8 h), resulting in reduced disease severity in the rhesus macaque model. This appears to simulate mild-to-moderate human MERS-CoV cases, making it difficult to extrapolate the outcome of this early intervention in severe human cases. Even though the authors recommended that combined IFN-α2b and ribavirin therapy should be considered as an early intervention therapy for MERS-CoV [115], we also need to keep in mind that, due to the limited effective therapeutic window of opportunity, broad spectrum antivirals might not be sufficient to treat severe MERS-CoV patients [122].

Resveratrol has been shown to inhibit various human viruses in vivo and in vitro, including influenza virus, Epstein–Barr virus, herpes simplex virus, respiratory syncytial virus, HIV-1, varicella zoster virus, enterovirus 71, human metapneumovirus, human rhinovirus 16, polyomavirus and cytomegalovirus ([123,124] for review). The antiviral effects of resveratrol are mainly associated with the inhibition of viral replication, protein synthesis, gene expression, and/or nucleic acid synthesis [123,124,125]. In an in vitro study, resveratrol was shown to significantly inhibit MERS-CoV infection, most likely due to the observed inhibition of MERS-CoV nucleocapsid (N) protein expression [126], a multifunctional protein essential for CoV replication [127]. Furthermore, resveratrol downregulated apoptosis induced by MERS-CoV, thereby prolonging cellular survival post-infection [126]. Although the beneficial roles of resveratrol in several viral diseases have been well documented, adverse effects have been also been reported, including increasing viral RNA replication during Hep-C virus infection in vitro (OR6 cells) [128], strong cytotoxicity in cultured cells [129], as well as enhanced HBV transcription and replication in vitro and in vivo [130]. Clearly, the antiviral potential of resveratrol in MERS-CoV infections needs to be studied more extensively, but, based on the various unintended negative effects, this needs to proceed with caution.

More recently, de Wilde et al. [131] reported that in an in vitro test, low-micromolar concentrations of alisporivir, a non-immunosuppressive cyclosporin A-analog, inhibit the replication of four different coronaviruses, including MERS-CoV. In this study, ribavirin was found to further potentiate the antiviral effect of alisporivir in the in vitro infection models, which warrants the further exploration of cyclophilin inhibitors as potential host-directed, broad-spectrum inhibitors of coronavirus replication [131].

3C-like protease (3CL^pro^)—analogous to picornavirus 3C protease (3C^pro^)—is functionally important in the CoV replication cycle [132] and is thus regarded as a validated drug target. Peptidomimetic inhibitors of enterovirus 3C^pro^ (6b, 6c and 6d) inhibited MERS-CoV 3CL^pro^ and in MERS-CoV-infected cells, the inhibitors showed antiviral activity by downregulating viral protein production in cells, as well as reducing release of infectious viral particles into culture supernatants. These compounds exhibited good selectivity index and should be investigated further as, not only an inhibitor of MERS-CoV replication and infections, but also as broad-spectrum antiviral activity drugs against other CoVs and picornaviruses [133]. Our laboratory has also previously screened the ZINC drugs-now library for candidates with potential anti-3CL^pro^ activity with a consensus high-throughput pharmacophore modelling and molecular docking approach. Molecular dynamics was used to confirm results obtained from structure-based techniques, resulting in a highly defined hit-list of 19 compounds, which represent valuable scaffolds that could be used as a basis for future anti-coronaviral inhibitor discovery experiments [50,134]. Even with all of these potential anti-MERS-CoV candidates, no experimental interventions have demonstrated significant benefit in acutely ill patients in a consistent or controlled manner. Therefore, supportive management, adapted from guidelines developed for SARS-CoV, has thus far been the mainstay of MERS-CoV treatment [135].

Because of the highly sophisticated immune evasion mechanisms of viral pathogens, human vaccine development remains a major challenge [136]. In addition, the development of safe and effective coronavirus vaccines has been even more challenging, being curtailed by major obstacles, including (1) coronavirus immunity often wanes rapidly; (2) individuals needing to be protected include the elderly; and (3) vaccines may exacerbate rather than prevent coronavirus lung immunopathology [137,138]. Various vaccines against MERS-CoV have been designed, one of which are currently being tested in clinical trials (Table 2). All of the MERS-CoV structural proteins could potentially induce neutralizing antibodies and protective responses. However, prior to identification of the major neutralizing antibody-inducing epitopes, inactivated viruses could be used in the production of first-generation vaccines; this is an easy first-response approach since it is relatively simple to produce whole killed virus particles [139]. With the many safety concerns associated with the production of inactivated vaccines [140,141,142], these types of vaccines must preferably be replaced by safer and more effective neutralizing epitope-based vaccines, as soon as the fragments containing the neutralizing epitopes are identified [139]. Current MERS-CoV vaccines provide effective protection in a few animal models [143,144,145,146,147].

## 7. Future Perspectives

The emergence of Middle East respiratory syndrome (MERS) and the discovery of the MERS coronavirus (MERS-CoV) in 2012 suggest that another SARS-like epidemic is occurring. Unlike the severe acute respiratory syndrome (SARS) epidemic, which rapidly disappeared in less than one year, MERS has persisted for over three years. More than 2000 cases of MERS have been reported worldwide, and the disease carries a worryingly high fatality rate of >30% [12]. While this number seems low, the virus remains a global threat due to its propensity to cause severe disease in patients with underlying medical conditions and its apparent ability to readily spread within hospital settings [148]. In addition, the pattern of MERS-CoV lineages is more consistent with the movement of infected livestock or animal products [171] and epidemiological evidence suggests that it is periodically introduced into human populations [172,173], which increases the risk for various future pandemics.

Even though the clinical outcomes of MERS-CoV infections are well documented, more comprehensive population-based studies are required to determine the involvement of MERS-CoV in other body systems. In addition, the continued development of technologies to routinely and accurately identify asymptomatic MERS-CoV infections will shed light on the true incidence of this virus in the human population. It would appear the MERS-CoV has been circulating in the human population for greater than one year without detection and suggests independent transmission from an unknown source. However, as discussed previously with regard to the emergence of severe acute respiratory syndrome coronavirus (SARS-CoV) in 2002, other evolutionary aspects, such as mutation rates and selection pressure, should be considered to understand the evolutionary dynamics of MERS-CoV [174,175,176,177]. Possibly different molecular clock rates of MERS-CoV in animal hosts and humans may also have to be taken into account. Similarly to the genomic evolution of influenza A viruses [178], MERS-CoV might experience different evolutionary courses in different hosts. To better understand these dynamics, the chain of MERS-CoV zoonotic transmissions should be further clarified [174].

As with other HCoVs, a detailed manipulation of the MERS-CoV genome to understand the role of the MERS-CoV viral genes in pathogenesis and replication, and for the subsequent development of MERS-CoV as a vaccine vector, is needed. The development of MERS-CoV full-length infectious clones [19,20,179] already allows for the systematic experimental study of the roles of the various corresponding MERS-CoV proteins, which should lead to a better understanding of the role of the viral genes in infectivity and pathogenicity [180]. This manipulation of the virus genome also provides a reverse genetics platform that could lead to the future development of MERS-CoV-based vector vaccines [181].

As a result of the increase in MERS spread, the WHO (World Health Organisation) and CDC (Center for Disease Control) have released various case definitions to allow for the likelihood of a pandemic threat to be reduced. Fever, pneumonia, and acute respiratory distress syndrome with a history of travel to the Arab Peninsula are some of the symptoms that are used to diagnose a MERS-CoV infection. Due to the increase in nosocomial infections, health care workers are also advised to be aware of any upper respiratory tract infections and exposure to MERS-CoV-positive individuals [182]. For the foreseeable future, important measures to prevent nosocomial outbreaks should include good compliance with appropriate personal protection equipment by health-care workers when managing patients with suspected and confirmed MERS-CoV infection, early diagnosis, prompt isolation of infected patients, and improvement of ventilation in health-care facilities [183,184].

## Figures and Tables

**Table 1:** Detection methods of MERS-CoV.

Method Used for Detection	1 Sensitivity/2 Specificity/3 Viral Target Gene	Reference
rtRT-PCR	1 Sensitivity for upE is 3.4 copies per reaction (95% confidence interval (CI): 2.5–6.9 copies) or 291 copies/mL of sample. 2 No cross-reactivity was observed with coronaviruses OC43, NL63, 229E, SARS-CoV, nor with 92 clinical specimens containing common human respiratory viruses. 3 Targeting regions upstream of the E gene (upE) or within open reading frame (ORF) 1b, respectively.	[75]
qRT-PCR ^#^	1 Sensitivity to widely used upE gene as well as a ORF1a&b was introduced 2 No false-positive amplifications were obtained with other human coronaviruses or common respiratory viral pathogens or with 336 diverse clinical specimens from non-MERS-CoV cases; specimens from two confirmed MERS-CoV cases were positive with all assay signatures. 3 Two novel signatures used one that targets the MERS-CoV N gene in combination with the upE test. The other a positive test to add to an efficient MERS-CoV kit.	[81]
RT-Sequence-Validated-LAMP Assays	1 Could detect 0.02 to 0.2 plaque forming units (PFU) (5 to 50 PFU/mL) of MERS-CoV in infected cell culture supernatants. 2 Did not cross-react with common human respiratory pathogens.	[88]
RT-LAMP	1 Capable of detecting as few as 3.4 copies of MERS-CoV RNA; Assay exhibited sensitivity similar to that of MERS-CoV real-time RT-PCR. 2 No cross-reaction to other respiratory viruses. 3 Assay designed to amplify the MERS-CoV gene	[80]
rt-RPA	1 Highly sensitive, is able to detect 10 MERS-CoV RNA copies with a more rapid detection time than MERS-RT-PCR. 2 No cross-reaction to other respiratory viruses including HCoVs. 3 Assay designed to amplify the partial nucleocapsid gene of MERS-CoV	[89]
mAb Test	1 Rapid detection and cost effective ELISA 2 High specificity used to detect the MERS-CoV nucleocapsid protein	[87]
Immuno-chromotagraphic tool	1 Highly sensitive, 2 No cross reactivity with other respiratory pathogens observed in vitro and in silico 3 Detects recombinant MERS-CoV N protein	[90]
Immunofluorescence Assay	1 Highly sensitive, antigen based detection 2 Cross reactivity seen with convalescent SARS patient (sera) 3 Assay used both whole virus and S1 portion of the spike protein	[91,92,93]
ppNT Assay	1 Highly sensitive, more sensitive that MNT test 2 Lack of MERS neutralizing activity indicated high specificity by this assay. No cross reactivity seen with SARS-CoV 3 Assay was designed for two different genes used: a codon optimized spike gene and a HIV/MERS pseudoparticle was generated	[94,95]
MNT Test	1 Highly sensitive; less so than ppNT assay 2 Highly specific, as SARS-CoV antigen was not detected compared to MERS-CoV. 3 Test designed to detect IgG antibodies generated when using the RBD of the S1 subunit of the spike gene	[94,96,97]
Protein Microarray	1 Highly sensitive assay using protein microarray technology to detect IgG and IgM antibodies 2 No cross reactivity seen with sera of patients that had been exposed to four common HCoVs. 3 Assay designed to use the S1 receptor-binding subunit of the spike protein of MERS and SARS as antigens.	[98]
One pot RT-LAMP	1 Capable of detecting four viral copies MERS within 60 min 2 No cross-reaction to the other acute respiratory disease viruses (influenza type A virus (H1N1 and H3N2), influenza type B virus, HCoV-229E, and human metapneumovirus) 3 Six sets of primers designed specifically to amplify the MERS-CoV genes	[99]
RT-iiPCR assays	1 Could detect 3.7 × 10^−1^ plaque forming units (PFU) of MERS-CoV in infected cell culture supernatants and sputum samples. 2 Viral nucleic acids extracted from infected cultures that contained HCoV-229E, HCoV-OC43, FIPV, influenza type A and B virus strains yielded negative results, indicating no cross reactivity. 3 Targeting regions upstream of the E gene (upE) or within open reading frame (ORF) 1b	[100]
Powerchek MERS Assay	1 95% limits of detection of assay for the upE and ORF1a were 16.2 copies/μL and 8.2 copies/μL, respectively. 2 No cross reactivity with other respiratory pathogens observed in vitro and in silico 3 Targeting regions upstream of the E gene (upE) or within open reading frame (ORF) 1b	[101]
acpcPNA-AgNP aggregation assay	1 Probe designed for targets makes this assay highly specific. Limit of detection found to be 1.53 nM 2 Cross reactivity with other CoVs was not evaluated 3 Synthetic oligonucleotides were designed to target MERS	[102]
mCoV-MS	1 Highly sensitive, multiplex PCR based to target specific genes in HcoVs 2 Cross reactivity with other respiratory pathogens was not evaluated 3 Targeting regions upstream of the E gene (upE) or within open reading frame (ORF) 1b	[103]
Duplex-RT-PCR method	1 Highly sensitive, simultaneous detection of MERS and SARS viruses. 2 Cross reactivity with other respiratory pathogens was not evaluated 3 Primers and probes that target the conserved spike S2 region of SARS-CoV, MERS-CoV, and their related bat CoVs were used	[104]

**rtRT-PCR:** Real-time reverse transcription polymerase chain reaction; **LAMP:** Loop-mediated isothermal amplification; **qRT-PCR:** Quantitative real-time reverse transcription polymerase chain reaction; **rtRPA:** reverse transcription isothermal Recombinase Polymerase Amplification; **mAb:** monoclonal Antibody; **ELISA:** Enzyme linked immunoabsorbent assay; **ppNT:** pseudoparticle neutralisation; **MNT:** microneutralisation; **RT-iiPCR:** reverse transcription-insulated isothermal PCR; **Powerchek:** PowerChek MERS assay; Kogene Biotech, Korea; **acpcPNA-AgNP:** DNA detection based on pyrrolidinyl peptide nucleic acid induced silver nanoparticle (colorimetric assay); **mCoV-MS:** MassARRAY matrix-assisted laser desorption/ionization time-of-flight mass spectrometry (MALDI-TOF MS) system; **N:** Nucleocapsid; ^#^ FDA approved (RealStar MERS-CoV RT-PCR kit 1.0, Altona Diagnostics GmbH, Hamburg, Germany).

**Table 2:** MERS-CoV vaccines developed (adapted from [135,148]).

Vaccine Categories	Target Antigen	Immunization	Animal Model	Immunogenicity	Stage of Development	Reference
Anti-MERS-CoV monoclonal antibodies	Surface (S) glycoprotein	Passive	marmosets	Animals developed pneumonia, high viral titre detected in lungs	Preclinical: in vivo, efficacy stage	[149,150,151]
Human polyclonal anti-MERS-CoV antibodies	Virus structural proteins	Passive	Ad5-hDPP4-transduced mouse	Nab developed to reduce viral titres post exposure	Preclinical: in vivo, efficacy stage	[152]
Inactivated virion vaccines	MERS-CoV	Active	hDPP4-transgenic mice	Nab produced without adjuvant, T-cell response not done	Preclinical: in vivo, efficacy stage	[153]
Live attenuated vaccines (deleted E protein; mutated in nsp14)	rMERS-CoV-∆E	Active	Not tested	Not indicated	Preclinical development: in vitro	[20]
Recombinant viral vectors (MVA, Adenovirus, Parainfluenza virus, Measles, Rabies)	S and SolS proteins	Active	Ad/hDPP4-mice Camels	Nab in mice, antigen specific humoral and in some case T cell immune responses	Preclinical: in vitro, efficacy stage	[145,154,155,156,157,158]
Replicon particles (e.g., Venezuelan (VRP-S)	S protein	Active	Ad/hDPP4-mice mice	Nab produced, mice developed progressive pneumonia with virus replication detected in airways	Preclinical: in vivo, efficacy stage	[110,159]
Subunit vaccines RBDs rRBDs RBDs-Fc rNTDs	S/S1protein with various amino acid residues	Active	-hDPP4-transgenic-Ad5-hDPP4 mice Rabbit NHPs	High mucosal and humoral immune response, strong Nab in mice and rabbits. Good T-cell response in mice. Tg-Mice protected from MERS-CoV	Preclinical: in vitro, efficacy stage	[147,160,161,162,163,164,165]
DNA vaccines	S protein	Active	NHP:Rhesus Macaques Camels Mice	Cellular immune response and Nab response in mice, NHPs and camels.	Phase 1 clinical trials	[166]
DNA prime/Protein-boost Vaccines	S and S1 protein	Active	NHP:Rhesus Macaques Mice	Nab response seen in mice and NHPs	Preclinical: in vitro, efficacy stage	[167]
VLPs	S, M, E	Active	NHP:Rhesus Macaques	Virus specific Nab and IgG antibody response against the RBD	Preclinical: in vivo, efficacy stage	[168]
Nanoparticle vaccine	S protein	Active	Mice	Nab with the presence of adjuvant (M1 and Alum)	Preclinical: in vivo, efficacy stage	[169,170]

**Ad:** Adenovirus; **Ad/hDPP4-mice:** mice transduced with hDPP4 in an adenovirus vector; **Alum**: aluminum hydroxide (adjuvant); **∆E:** truncated envelope protein, **hDPP4:** human dipeptidyl peptidase 4; **M1:** matrix protein 1 (adjuvant); **MERS-CoV:** Middle East Respiratory Syndrome Coronavirus; **M:** membrane protein; **MVA:** modified vaccinia virus Ankara; **N:** nucleocapsid protein; **Nab:** neutralizing antibody; **NHP:** non-human primates; **rMERS-CoV:** recombinant Middle East respiratory syndrome coronavirus; **rNTD:** recombinant N-terminal domain; **RBD:** receptor-binding domain; **rRBD:** recombinant RBD; **RBD-Fc:** RBD fused to the human IgG antibody crytallizable fragment; S: spike protein; **S1:** S1 domain of the spike protein, **SolS:** spike protein lacking transmembrane domain; **Tg-mice**: transgenic mice; **VRP:** virus replicon particle; **VLP’s:** virus like particles.
